# Gangrene of the Scrotum—Autoplasty—Use of Collodion

**Published:** 1850-01

**Authors:** 


### SURGERY.

*Gangrene of the Scrotum—Autoplasty—Use of Collodion.*—Although gangrene of the scrotum, giving rise to more or less complete exposure of the testicles, has frequently presented itself to the practice of surgeons, few efforts have been made to remedy this distressing accident in a scientific manner. In some cases the whole of the front of the scrotum has been destroyed, and, as cicatrization advances, the wound contracts behind the testicles, thus increasing the exposed condition of these organs.

In such extreme cases surgeons have been usually content to leave the cure to nature, when the testicle is finally covered by a thin and imperfect cicatrix. M. Malgaigne, one of the most rising young surgeons of the French school, has acted differently. In two cases of the kind alluded to, he separated the adherent edges of the wound from the testicle, formed a cavity behind in what remained of the scrotum, and united the edges in front of the testicle with the twisted suture. These two cases were completely successful. The following case, somewhat analogous, is a striking example of the resources of surgical skill under the most unfavorable circumstances:—

A soldier of the 32d Regiment was admitted into the hospital with complete exposure of both testicles, the consequence of gangrene of the scrotum after small-pox. The wound was cicatrising, but the loss of substance had been so great, that the cicatrix, in contracting, pushed the testicles forward, and thus exposed them more and more every day. It was, therefore, resolved to imitate the practice of M. Malgaigne, and endeavor to unite the remnant of the envelope in front of the organ. The patient was placed under the influence of chloroform, and within a minute fell into a deep sleep. The skin was now separated from the lateral and inferior portions of the cicatrix, and the dissection carried back sufficiently to allow of the integument being brought forward so as to cover the testicle completely. The edges of the wound were then rendered smooth with the scissors, brought forward and united in front along the median line, by five points of the twisted suture. A small branch of the pudic artery was tied. The usual dressings, supported by a suspensory bandage, were next applied, and the patient replaced in bed. On the fourth day after the operation, it was found that the needles had cut through the skin; that the flaps showed no disposition to unite, and, moreover, were so attenuated as to prevent the use of diachylon straps. Under these circumstances, the Surgeon had recourse to the use of collodion. Two small pieces of linen, cut into the shape of two cutaneous flaps, were imbibed with collodion, and then applied along the external surface of the wound, on both sides, from above, downwards. Six short ligatures were then glued, in pairs, and opposite each other, with the same substance to the linen bands,—one pair just under the penis; the other at the middle of the wound; the third near its inferior angle. On tying together these ligatures, the edges of the wound were brought together, completely in front of the testicles. This apparatus was allowed to remain undisturbed for six days. On removing it, the flaps were found adherent to the subjacent surfaces, and also by their edges, except at the superior and inferior angles. Here were some exuberant granulations, which were touched with the nitrate of silver. A fresh dressing, similar to the former one, was now applied, and allowed to remain for four days longer. At the expiration of this period, the whole of the wound was completely united, with the exception of a few lines at the lower angle, and even this cicatrised on the thirtieth day. The scrotum then presented, in every respect, a normal aspect.

The practice adopted in the above case is well worthy of imitation, under analogous circumstances. It would, however, appear advisable to have recourse to the collodion at once, instead of wasting time by an endeavor to unite the edges of the wound with the twisted suture. In cases of this kind, the surface of the exposed testicle is almost always in a state of suppuration, and it is extremely difficult, if not impossible, to make the sound skin adhere to this suppurating tissue through the medium of suture.—*Medical Times.*

